# Atropine augments cardiac contractility by inhibiting cAMP-specific phosphodiesterase type 4

**DOI:** 10.1038/s41598-017-15632-x

**Published:** 2017-11-09

**Authors:** Ruwan K. Perera, Thomas H. Fischer, Michael Wagner, Matthias Dewenter, Christiane Vettel, Nadja I. Bork, Lars S. Maier, Marco Conti, Juergen Wess, Ali El-Armouche, Gerd Hasenfuß, Viacheslav O. Nikolaev

**Affiliations:** 1 Institute of Experimental Cardiovascular Research, University Medical Center Hamburg-Eppendorf, Hamburg, Germany; 2 Clinic of Cardiology and Pulmonology, Heart Research Center Göttingen, Georg August University Medical Center, Göttingen, Germany; 3 Institute of Pharmacology and Toxicology, Technical University of Dresden, Dresden, Germany; 4 Institute of Experimental and Clinical Pharmacology and Toxicology, Mannheim Medical Faculty, Heidelberg University, Mannheim, Germany; 5 DZHK, German Center for Cardiovascular Research, partner sites Hamburg/Kiel/Lübeck and Göttingen, Hamburg, Germany; 6 Department of Internal Medicine II, University Hospital Regensburg, Regensburg, Germany; 7 Department of Obstetrics, Gynecology and Reproductive Sciences, University of California, San Francisco, United States; 8 Molecular Signaling Section, Laboratory of Bioorganic Chemistry, National Institute of Diabetes and Digestive and Kidney Diseases (NIDDK), Bethesda, Maryland USA

## Abstract

Atropine is a clinically relevant anticholinergic drug, which blocks inhibitory effects of the parasympathetic neurotransmitter acetylcholine on heart rate leading to tachycardia. However, many cardiac effects of atropine cannot be adequately explained solely by its antagonism at muscarinic receptors. In isolated mouse ventricular cardiomyocytes expressing a Förster resonance energy transfer (FRET)-based cAMP biosensor, we confirmed that atropine inhibited acetylcholine-induced decreases in cAMP. Unexpectedly, even in the absence of acetylcholine, after G-protein inactivation with pertussis toxin or in myocytes from M_2_- or M_1/3_-muscarinic receptor knockout mice, atropine increased cAMP levels that were pre-elevated with the β-adrenergic agonist isoproterenol. Using the FRET approach and *in vitro* phosphodiesterase (PDE) activity assays, we show that atropine acts as an allosteric PDE type 4 (PDE4) inhibitor. In human atrial myocardium and in both intact wildtype and M_2_ or M_1/3_-receptor knockout mouse Langendorff hearts, atropine led to increased contractility and heart rates, respectively. *In vivo*, the atropine-dependent prolongation of heart rate increase was blunted in PDE4D but not in wildtype or PDE4B knockout mice. We propose that inhibition of PDE4 by atropine accounts, at least in part, for the induction of tachycardia and the arrhythmogenic potency of this drug.

## Introduction

The autonomic nervous system regulates functions of various organs via the sympathetic and parasympathetic neurotransmitters norepinephrine and acetylcholine (ACh). Once released from nerve terminals, they activate adrenergic and muscarinic G-protein coupled receptors on the membranes of target cells. In the heart, sympathetic innervation augments contractility by a β-adrenergic receptor (β-AR)-mediated increase in intracellular levels of the second messenger 3′,5′-cyclic adenosine monophosphate (cAMP). Conversely, ACh released from parasympathetic nerves acts on muscarinic M_2_-receptors, resulting in decreased intracellular cAMP, heart rate and contractility^1–3^.

Atropine, which is on the WHO List of Essential Medicines, is a non-selective muscarinic receptor inhibitor used to treat acute sinus node dysfunction associated with bradycardia, complete atrioventricular block, and organophosphate and beta-blocker poisoning. Therefore, atropine is widely used for resuscitation and emergency cardiovascular care^4^ as well as for critical care intubation in neonates and to decrease salivation prior to some surgeries^5^. In the heart, atropine blocks the inhibitory effect of ACh on heart rate and contractility, potentially also leading to tachyarrhythmias^6^. These and other prominent effects of atropine have been exclusively attributed to its antagonism at muscarinic receptors^7,8^. However, paradoxical actions of this drug on cardiovascular system and a plethora of side-effects ranging from anticholinergic up to less well-explicable nausea and paradoxical bradycardia seem to rely on more than one classical mechanism of action^6^.

Here, we use various *in vitro* and *in vivo* techniques to test the hypothesis that atropine can inhibit the activity of cAMP hydrolysing phosphodiesterases (PDEs), thereby increasing intracellular cAMP levels. We found that atropine, independently of its effect on muscarinic receptors, can inhibit PDE4 activity, leading to augmented cardiac contractility after β-adrenergic stimulation. This new receptor-independent mechanism may explain many of the pharmacological actions and side-effects of this classical cardiovascular drug.

## Results and Discussion

### Atropine increases cAMP independently of M_1/2/3_-muscarinic receptors

**Figure 1:**
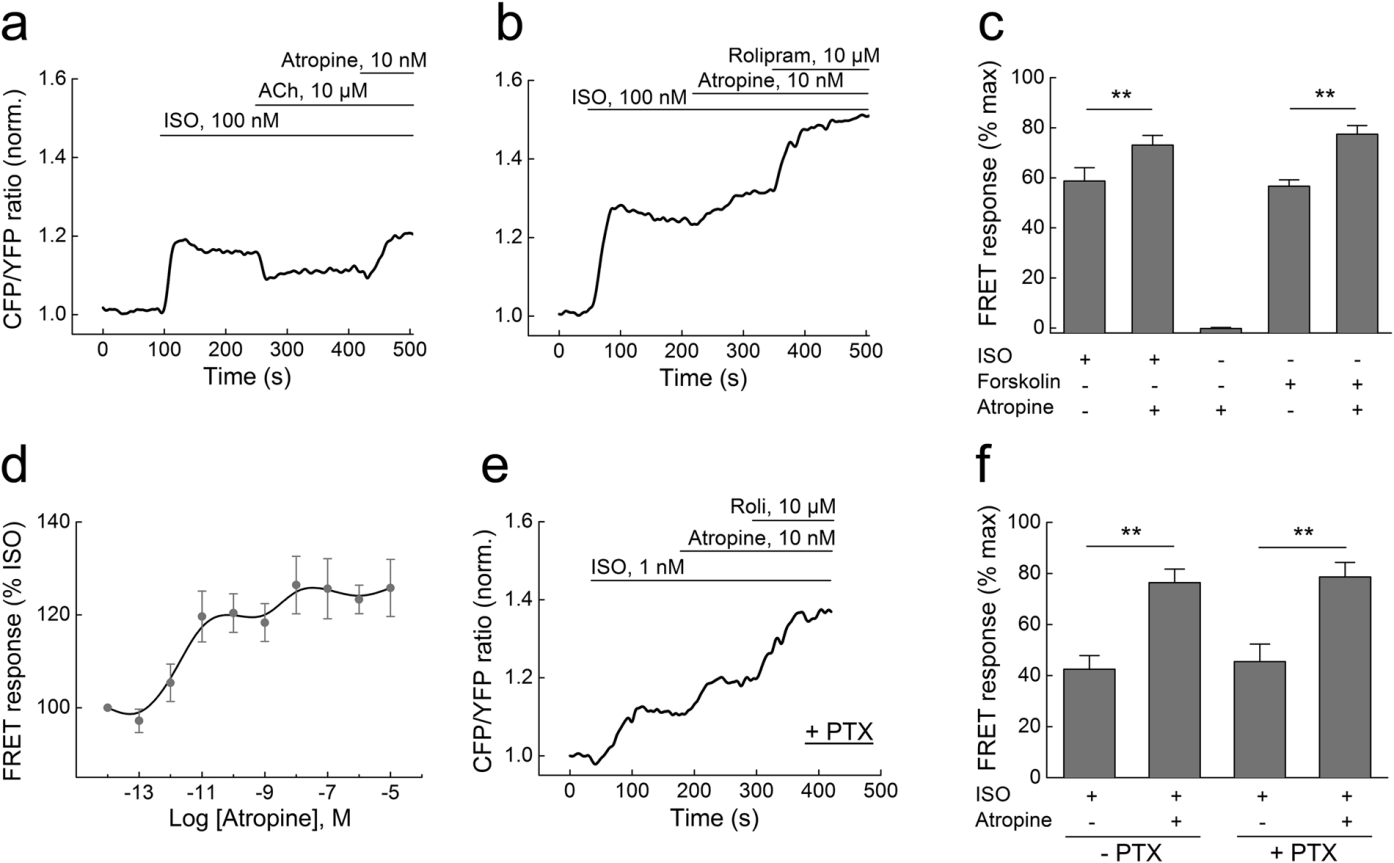
Single-cell FRET analysis of intracellular cAMP levels in adult ventricular mouse cardiomyocytes transgenically expressing the Epac1-camps sensor. (**a**) The β-AR agonist isoproterenol (ISO, 100 nM) increases intracellular cAMP (indicated by normalized CFP/YFP ratio), and this response is partially reversed by acetylcholine (ACh, 10 µM). The ACh effect is completely blocked by atropine (10 nM), and the cAMP levels are even further increased compared to the steady-state reached after ISO stimulation. CFP, enhanced cyan fluorescent protein; YFP, enhanced yellow fluorescent protein. (**b**) Atropine increases cAMP levels beyond the plateau reached after ISO stimulation. They can be further elevated by the selective PDE4 inhibitor rolipram (10 µM). Data in **a** and **b** are representative traces, quantification is shown (**c**) as mean ± s.e.m. (n = 5–8). The data are presented as a % of the maximal FRET response reached after ISO/rolipram or forskolin/rolipram stimulation. (**d**) Concentration-response dependency of atropine on cAMP levels in cardiomyocytes after ISO prestimulation. (**e**) Inhibition of G_i_-proteins with pertussis-toxin (1.5 µg/ml for 7–8 h) does not affect the atropine mediated stimulation of cAMP. Here we used 1 nM ISO, since 100 nM lead to a complete saturation in PTX-treated cells. Representative experiment (n = 5). Quantification of the FRET ratio changes is shown in (**f**). Here and in C: **differences are statistically significant at, p < 0.01 by one-way ANOVA.

To elucidate the exact molecular mechanisms of atropine action in the heart, we studied its effects in cardiomyocytes isolated from mice expressing the Förster resonance energy transfer (FRET)-based cAMP-sensor Epac1-camps^9,10^. Stimulation of these cells with the β-AR-agonist isoproterenol (ISO) leads to an increase of intracellular cAMP which can be partially (~50%) reversed by ACh^11^. As expected, application of atropine (10 nM) after ACh fully blocked this ACh effect, while application of atropine alone, even at high concentrations (10 µM), had no effect on cAMP levels (Fig. 1a, Supplementary Figure 1a). Unexpectedly, when applied after ISO and in the absence of ACh, atropine (10 nM) potentiated the β-AR-induced cAMP response (Fig. 1b,c **)**. The same atropine activity was observed in cells prestimulated with forskolin, a direct adenylyl cyclase activator (Fig. 1c, Supplementary Figure 1b). We next analyzed the concentration-response dependency of this atropine effect in cardiomyocytes and found that the maximal effect was achieved already at ~10 nM (Fig. 1d), which is in the therapeutically relevant concentration range of this drug^6,7^.

The stimulatory effect of atropine on cAMP levels following ISO treatment is compatible with data from frog and rat ventricular myocytes where atropine was shown to stimulate L-type calcium channel currents, presumably via a G-protein-dependent mechanism involving atropine binding to muscarinic receptors^12^. Of all five muscarinic receptor subtypes, cardiomyocytes predominantly express the M_2_-receptor which is coupled to inhibitory G-proteins of the G_i_ family^3^. In addition, M_1/3_-receptors have been shown to be functional in murine and rat hearts^13–15^. We first inactivated G_i_-proteins by pertussis-toxin (PTX) and then analyzed the effects of atropine on cAMP levels. Interestingly, treatment with PTX completely abolished the effect of ACh (Supplementary Figure 1c,d) but did not affect the atropine-mediated increase in cytosolic cAMP levels after ISO stimulation, indicating that this effect does not involve G_i_-proteins (Fig. 1e,f). We also observed a well-documented increase of sensitivity to ISO after PTX treatment which might be due to off-target effects of this toxin^16^. To further test the hypothesis that atropine can mediate cardiac effects independent of muscarinic receptor blockade, we isolated ventricular cardiomyocytes from M_2_- or M_1/3_-receptor knockout mice^17,18^ and expressed the cAMP-FRET sensor in these cells by adenoviral gene transfer. The lack of these three muscarinic receptor subtypes had no effect on the ability of atropine to enhance cAMP responses (Supplementary Figure 2).

### Atropine inhibits the cAMP-specific phosphodiesterase PDE4

**Figure 2:**
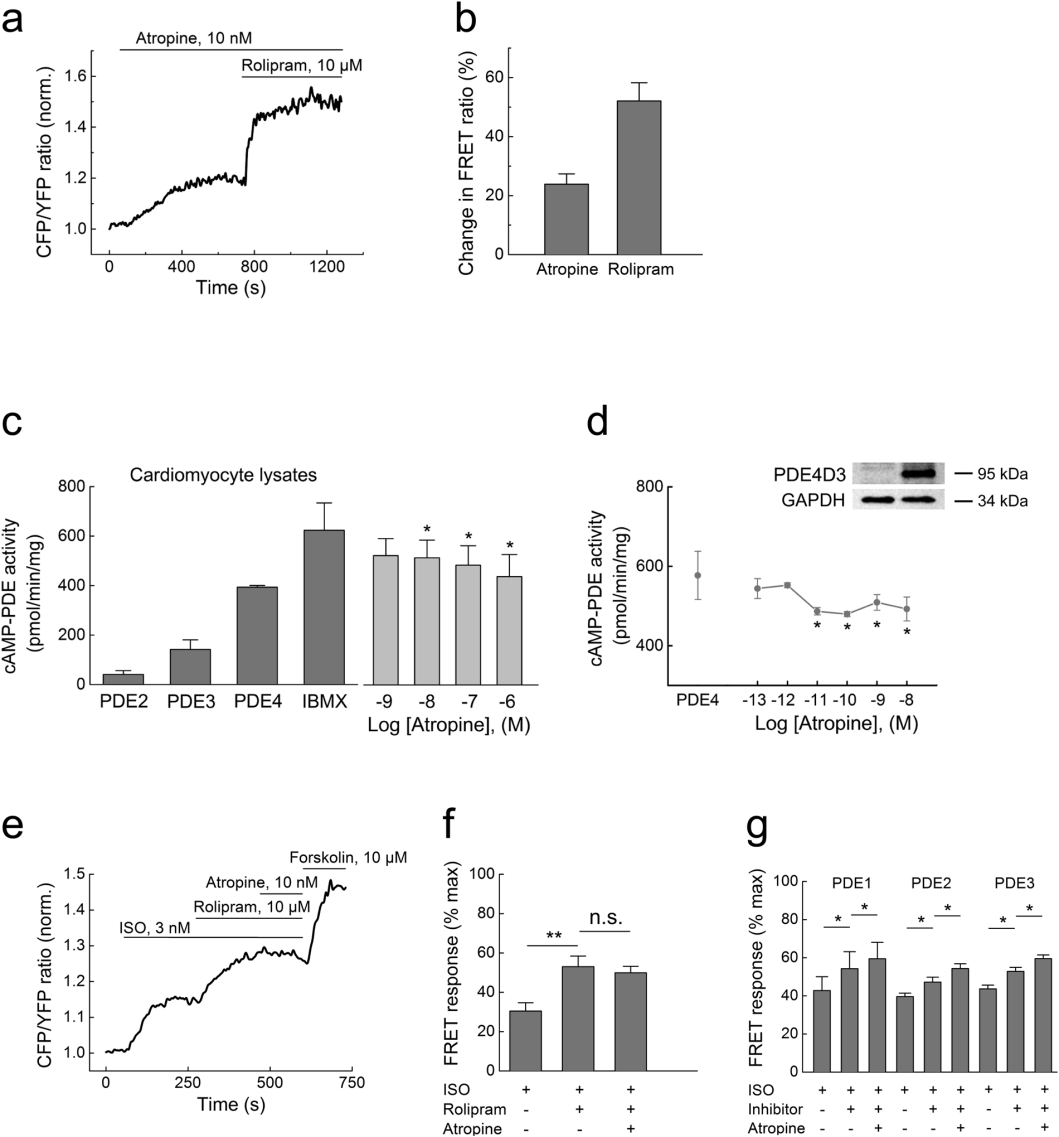
Atropine inhibits PDE4 activity. (**a**) Single-cell FRET analysis of PDE4 inhibition in HEK293A cells expressing the Epac1-camps-PDE4 sensor. Cells were prestimulated with 1 µM ISO for 3 min before adding atropine (10 nM) and rolipram (10 µM) to pre-elevate intracellular cAMP levels. (**b**) Quantification of the data shown in **a** as a % change of the FRET ratio in response to atropine along with maximal effects measured by these sensors with respective inhibitors, mean ± s.e.m. (n = 6). Atropine inhibits cAMP-PDE activity in cardiomyocyte lysates (**c**) and recombinant PDE4D3 from transfected HEK293 cells (**d**), as measured by a classical *in vitro* PDE assay (n = 3–5). The basal PDE activity is represented by the “IBMX” bar in **c** or “PDE4” bar (rolipram-sensitive activity of PDE4D3 transfected minus vector-transfected control [Co] cell lysates, a representative immunoblot for PDE4D3 is shown) in (**d**.) *p < 0.05 compared to basal PDE activity by one-way ANOVA. (**e**) Preincubation of cardiomyocytes with 10 µM rolipram prevents the atropine (10 nM) mediated increase of cAMP after ISO stimulation (3 nM to avoid sensor saturation by rolipram). Representative experiment (n = 6), data analysis is in **f**. (**g**) preincubation of cells with 30 µM 8-MMX to block PDE1 (n = 6), 100 nM of the PDE2 inhibitor BAY 60–7550 (n = 6) or 10 µM of the PDE3 inhibitor cilostamide (n = 7) under the same experimental conditions (except for 100 nM ISO used to prestimulate cAMP levels) did not abolish the effect of atropine. *p < 0.05, **p < 0.01, n.s. – not significant by paired t-test.

The stimulatory effect of atropine on cAMP levels was reminiscent of the activity of PDE inhibitors. For example, the PDE4 inhibitor rolipram similarly increased ISO-stimulated cardiomyocyte cAMP levels, albeit with a greater efficacy (Fig. 1b). Therefore, we tested the hypothesis that atropine can directly inhibit PDEs. Cardiomyocytes express at least five families of these enzymes, PDE1-5, which selectively degrade either cAMP (PDE4), cGMP (PDE5) or both cyclic nucleotides (PDE1-3)^19–22^. Recently, we introduced FRET biosensors which can measure cAMP or cGMP in the vicinity of various PDEs, thereby directly reporting PDE inhibition by various compounds in intact cells^23^. These sensors are comprised of PDE sequences fused to Epac1-camps or to the cGMP sensor cGES-DE2 and respond to PDE inhibitors with a change of FRET (Supplementary Figure 3a). In addition to previously described sensors for PDE3-5, we developed a new biosensor construct to measure PDE1 inhibition and performed experiments using such sensors in HEK293A cells. Atropine showed strong FRET responses at PDE4, while the responses related to PDE1, PDE3 and PDE5 inhibition were not affected, even at very high atropine concentrations (Fig. 2a,b and Supplementary Figure 3b–d). The effect of atropine on the FRET response detected with the Epac1-camps-PDE4A1 sensor was much more pronounced than the effect of other clinically relevant anticholinergic drugs such as ipratropium and darifenacin used at saturating concentrations (Supplementary Figure 3f).

Next, we investigated whether atropine can directly block PDE activity using a classical *in vitro* activity assay. In heart lysates, atropine inhibited cAMP-hydrolyzing PDE activity already at low nanomolar concentrations (Fig. 2c). Since FRET experiments pointed toward possible involvement of PDE4, we performed *in vitro* assays with lysates from HEK293A cells overexpressing the functionally relevant cardiac PDE4D3 isoform. We found that atropine could inhibit PDE4D3 activity, though with considerably lower efficacy than rolipram, a selective PDE4 inhibitor, which completely blocked PDE4 activity (Fig. 2d). This observation may be indicative of an unusual mechanism of atropine action on PDE4 enzymes, which is different from that of classical competitive PDE4 inhibitors. We therefore tested the possibility that atropine might inhibit PDE4 activity in an allosteric manner. Indeed, in FRET and *in vitro* assays, atropine treatment led to an increase of potency for the competitive inhibitor rolipram, strongly supporting this hypothesis (Supplementary Figure 4). Importantly, preincubation of cardiomyocytes with rolipram but not with PDE1, 2 or 3 inhibitors completely abolished atropine-induced increases of cAMP after β-adrenergic stimulation, further confirming that atropine acts by inhibiting PDE4 (Fig. 2e–g).

cAMP responses to atropine reached their steady state levels within several minutes but their concentration-dependency was clearly biphasic (see Fig. 1d). Although plasma membrane is permeable to atropine to a certain extent under physiological pH^24,25^, the biphasic shape of this curve and difference in kinetics of atropine effects between cardiomyocytes and HEK293A cells (compare Figs 1b and 2a) suggest that other mechanisms beyond passive diffusion might be involved. In addition, organic cation transporters, especially OCT3 that is expressed in the heart but barely detectable in HEK293 cells can facilitate active atropine uptake into cells^26^. We therefore tested the non-selective OCT blocker MPP + in cardiomyocytes and found that it completely abolished the rapid effect of atropine on cAMP levels (Supplementary Figure 5a,b **)**. Conversely, stable expression of OCT3 in HEK293 cells led to a more robust atropine response (Supplementary Figure 5c), supporting the hypothesis that atropine can be actively transported into the cell where it inhibits PDE activity.

### PDE4 inhibition is involved in atropine induced positive inotropic and chronotropic effects *in vitro* and *in vivo*

**Figure 3:**
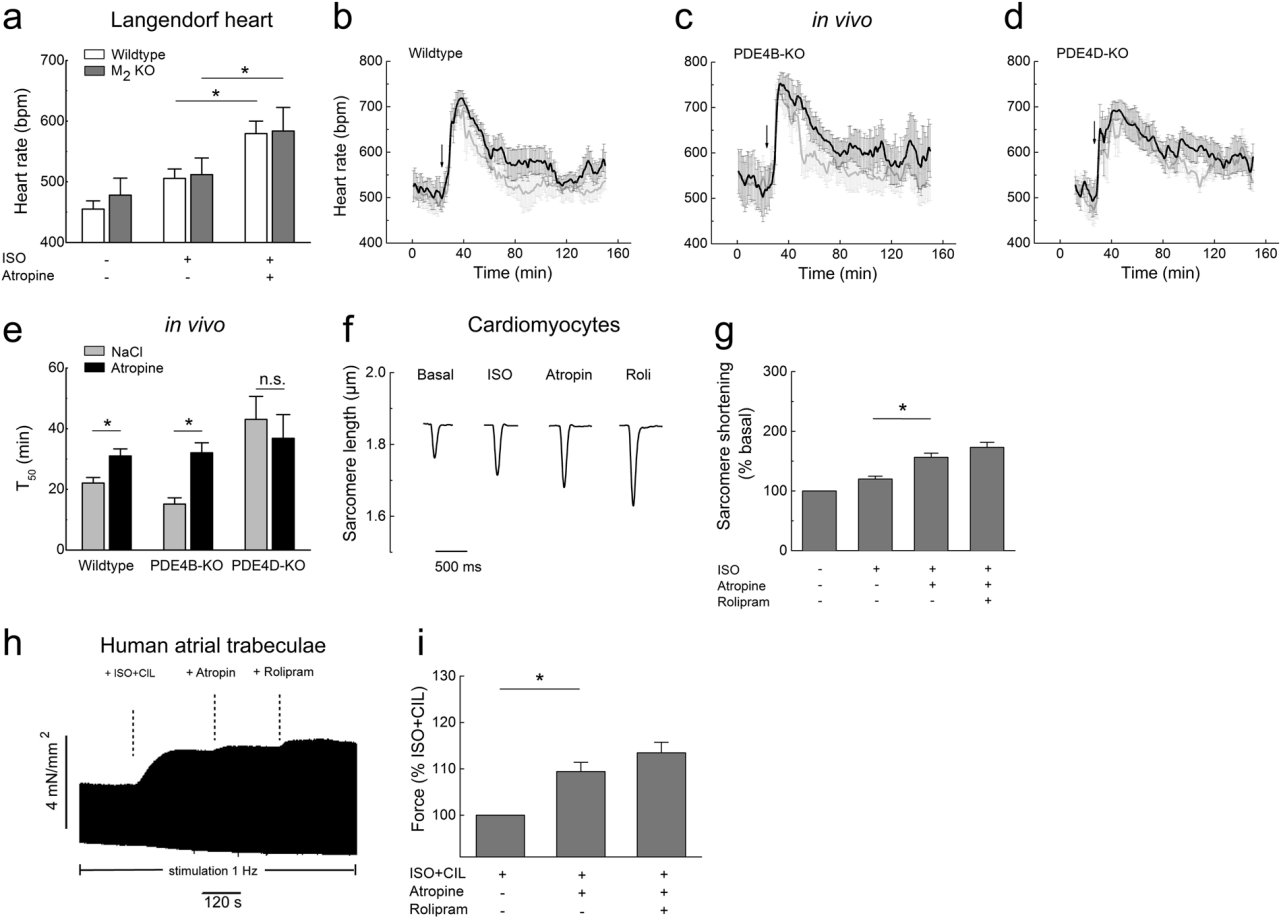
Atropine augments cardiac function in PDE4 dependent and muscarinic receptor independent manner. (**a**) Heart rate measurements in wildtype and M_2_-receptor knockout Langendorff hearts perfused with ISO alone (10 nM) or with ISO plus atropine (10 nM). Atropine applied after ISO significantly increases the beating frequency in both genotypes (n = 5). *p < 0.05, by paired t-test. (**b–e**) Averaged heart rate tracings and T_50_ values obtained from *in vivo* telemetry experiments in wildtype vs PDE4B or PDE4D knockout mice injected with atropine (0.5 mg/kg, denoted by arrow, black traces) or saline (NaCl, grey traces) control indicate that PDE4D but not PDE4B is involved in the hydrolysis of cAMP which regulates the duration of atropine-induced heart rate increase. T_50_ was defined as the duration of the increase in heart rate measured from half-maximal increase to half-maximal return to baseline. Number of mice used was 8, 5 and 4 for wildtype, PDE4B-KO and PDE4D-KO, respectively. (**f**) Representative traces from single cardiomyocyte contractility measurements by edge-detection show a positive inotropic effect of atropine (10 nM) applied after ISO (3 nM). Quantification is in (**g**), n = 10–13. In (**e**) and (**g**), *denotes significant differences p < 0.05 by one-way ANOVA. n.s. – not significant. (**h**) Original representative trace showing the effect of atropine on the developed force of contraction in human right atrial trabeculae. Atropine increases the force of contraction in trabeculae which were prestimulated with 1 nM ISO and 10 µM cilostamide (CIL). (**i**) Quantification of the contractility data. The data for each experiment were normalized to the force developed after prestimulation with ISO plus CIL. Mean ± s.e.m. (n = 5).*The differences are statistically significant (p < 0.05, by paired t-test).

What are the functional implications of this atropine effect on cardiac function? It is well accepted that atropine can induce tachyarrhythmias as a frequent and prominent side-effect *in vivo*, which has been mainly attributed to a decreased parasympathetic tone. We studied the effect of atropine on heart rate in explanted perfused mouse hearts (*ex vivo* Langendorff preparation) which lack any nervous innervation. As expected, a low dose of ISO increased the basal heart rate by ~10%. When atropine was applied after ISO, this response was greatly augmented (Fig. 3a), indicative of the positive chronotropic effect of atropine after β-adrenergic stimulation. Strikingly, this effect was preserved in M_2_- and M_1/3_-receptor knockout mouse hearts, further corroborating its receptor-independent nature (Fig. 3a, Supplementary Figure 6). In contrast, atropine applied alone had no effect on heart rate in this preparation (383 ± 42 at basal vs. 367 ± 46 bpm after 10 nM atropine alone; wildtype hearts, means ± s.e.m., n = 4, P = 0.83 by paired t-test).

To investigate the potential functional importance of PDE4 inhibition by atropine *in vivo*, we studied its effect on heart rate using telemetry in awake wildtype, PDE4B and PDE4D knockout (KO) mice. Interestingly, atropine induced an increase in heart rate, and this increase was significantly prolonged in wildtype and PDE4B but not in PDE4D KO mice, as compared to saline (NaCl) injected controls (Fig. 3b–e). This observation suggests that inhibition of PDE4D by atropine has functional effect on heart rate *in vivo*. Moreover, the prolongation of saline effect in PDE4D-KO compared to wildtype and PDE4B-KO mice (p = 0.004 and p = 0.017, respectively, by one-way ANOVA) indicates that PDE4D might be the major isoform for heart rate regulation under adrenergic stress. Since the PDE4A, B and D subfamilies share high sequence similarity, the observed lack of an atropine effect in PDE4D KO mice only is probably due to the fact that PDE4D is most important for heart rate regulation. When we analysed the effect of atropine on cAMP levels in single PDE4B and PDE4D KO cardiomyocytes, both genotypes indeed showed blunted effects of atropine (Supplementary Figure 2b).

Myocardial cAMP augmentation should not only lead to an increase in heart rate but also to increased contractile force. Sarcomere shortening measurements in isolated ventricular cardiomyocytes showed that atropine also enhanced the positive inotropic effect of ISO (Fig. 3f,g). This experiments support the concept that even in absence of parasympathetic innervation in isolated cardiomyocytes or explanted hearts, atropine increases cAMP levels and cardiac contractility by inhibiting PDEs.

In human atria, PDE4 accounts for only ~15% of the cAMP-specific PDE activity, whereas PDE3 represents the major PDE family^27,28^. Nevertheless, PDE4 plays an important protective role against atrial arrhythmias, and its effects on cAMP levels can be unmasked when PDE3 is inhibited^29^. We measured force of contraction in trabeculae isolated from human right atria. While atropine alone or atropine treatment after ISO prestimulation did not affect force of contraction (data not shown), application of atropine to trabeculae pretreated with ISO and the PDE3 inhibitor cilostamide substantially increased contractility which was further augmented by rolipram (Fig. 3h,i). This finding confirms that the positive inotropic effect of atropine is also relevant for the human heart and results from PDE4 inhibition.

In summary, we show that the clinically relevant drug atropine does not only block muscarinic receptors but also directly inhibits the enzymatic activity of PDEs, in particular the cAMP-specific PDE4. This new mechanism accounts for increased cAMP levels in cardiomyocytes which might play a crucial role in mediating various side-effects of atropine, especially arrhythmogenesis. We show that PDE inhibition by atropine promotes an increase in intracellular cAMP, which in turn leads to an elevated heart rate and increased contractility. This effect of atropine is clearly independent of M_1/2/3_-muscarinic receptors and does not involve its classical anticholinergic activity. The atropine-mediated increase in contractility by PDE4 inhibition is predicted to be especially important under adrenergic stress, which occurs either due to increased endogenous catecholamine levels in heart failure^8^, severe infections, myocardial infarction or during diagnostic procedures such as the dobutamine atropine stress echocardiography in a perioperative setting^30^. Since the stimulatory effect of atropine on cAMP production is only observed under catecholamine stress, it can be expected that therapeutically used β-blockers might effectively counteract atropine-induced arrhythmias associated with PDE inhibition.

## Methods

### FRET-based analysis of cAMP in cardiomyocytes

FRET measurements were performed in freshly isolated adult mouse ventricular myocytes, adenovirally transduced mouse cardiomyocytes (for 40–48 h with Epac2-camps adenovirus at multiplicity of infection 300)^31^ or transfected HEK293A cells as previously described^32^. Briefly, adult mouse ventricular myocytes were isolated from 8–20 week old mice using retrograde perfusion with enzymatic digestion. Hearts were Langendorff perfused at 37 °C with 3.5 ml/min of calcium-free perfusion buffer (in mM: NaCl 113, KCl 4.7, KH_2_PO4 0.6, Na_2_HPO_4_ × 2H_2_O 0.6, MgSO_4_ × 7H_2_O 1.2, NaHCO_3_ 12, KHCO_3_ 10, HEPES 10, Taurine 30, 2,3-butanedione-monoxime 10, glucose 5.5, pH 7.4) for 3 min followed by 30 ml digestion buffer containing liberase DH (0.04 mg/ml, Roche), trypsin (0.025%, Gibco) and 12.5 µM CaCl_2_. Cells were sedimented and gradually recalcified up to 1 mM of extracellular calcium before plating onto laminin-coated round glass coverslides. For FRET imaging, coverslides were mounted into Attofluor chamber (Life Science Technologies), washed once and maintained in the FRET buffer (in mM: 144 NaCl, 5.4 KCl, 1 MgCl_2_, 1 CaCl_2_, 10 HEPES, pH 7.3). Next, cell chamber was placed onto inverted fluorescent microscope (Nikon Ti) equipped with 440 nm pE-100 light source (CoolLED), DV2 DualView for CFP and YFP (Photometrics) and Orca 03 G camera (Hamamatsu). FRET imaging data were acquired using Micro-Manager 1.4 software (University of California San Francisco) and analysed using NIH Image J and Origin 8.5 software. CFP and YFP intensity values were corrected from the bleedthrough of the donor fluorescence (CFP) into the acceptor (YFP) channel to calculate the corrected CFP/YFP ratios.

### FRET analysis of PDE inhibition

To monitor inhibition of various PDE families by atropine, previously developed FRET-based sensors for human PDE3A, PDE4A1 and PDE5A were used^23^. In addition, we developed a new Epac1-camps-PDE1A sensor to monitor the inhibition of PDE1A. To generate this construct, the mouse PDE1A sequence was fused in frame to the C-terminus of Epac1-camps via a BamHI restriction site and a helical linker MPLVDFFC.

### *In vitro* PDE assays

Freshly isolated cardiomyocytes or HEK293A cells transfected with the PDE4D3 plasmid were lysed and processed for *in vitro* measurement of cAMP-PDE hydrolyzing activity following the standard method by Thompson and Appleman in presence of 1 µM cAMP as a substrate, as previously described^33,34^. Contributions of individual PDE families were calculated from the effects of 100 nM BAY (PDE2), 10 µM cilostamide (PDE3), 10 µM rolipram (PDE4), and 100 µM IBMX (unselective inhibitor). Alternatively, PDE assay was also performed using a recombinant PDE4A protein (P92-31G, Biozol, Eching, Germany) and the PDE-Glo^TM^ phosphodiesterase assay (Promega) according to the manufacturer’s protocol (Supplementary Figure 4). Samples were incubated for 15 min at 30 °C with various concentrations of rolipram in the presence or absence of atropine. These conditions led to a hydrolysis of ~50% of cAMP in the absence of inhibitors.

### Heart rate measurements

Mice were sacrificed by cervical dislocation. M_2_-muscarinic receptor knockout^17^ or M_1/3_-receptor double knockout^18^ and respective wildtype control animals were used. Hearts were rapidly explanted and subjected to Langendorff perfusion with the Krebs-Henseleit solution (in mM: 118 NaCl, 4.7 KCl, 1.2 KH_2_PO_4_, 1.25 MgSO_4_, 24 NaHCO_3_, 1.25 CaCl_2_ and 11.1 glucose; oxygenated with 95% O_2_ and 5% CO_2_) at 37.5 °C with a constant flow rate of 3.5 ml/min. Heart beats were detected with a custom made electrode and analyzed with Powerlab Chart 5 software (ADInstruments). Hearts were perfused for 3–5 minutes to reach stable baseline and then stimulated with 10 nM ISO for 3–5 min. After reaching the plateau phase, atropine was added for another 3–5 min.

### Single-cell contractility measurements

Freshly isolated ventricular cardiomyocytes were plated onto laminin-coated glass coverslides. Contraction amplitude was measured using the edge-detection method (IonOptix) at 1 Hz pacing frequency^35^.

### *In vivo* telemetry in awake mice

All animal experiments were performed according to institutional and governmental guidelines. Approvals for animal protocols were obtained from “Landesdirektion Sachsen” and the “Niedersächsische Landesamt für Verbraucherschutz und Lebensmittelsicherheit“. PDE4B and PDE4D knockout mice^36,37^ and corresponding wildtype control littermates were kept on C57Bl/J;FVB/N1 background. For implantation of ECG-transmitters (ETA-F10, DSI), mice were anaesthetized with 2% isoflurane. The ECG-transmitter was implanted subcutaneously to the back of the mouse, the negative electrode was fixed to the right pectoralis fascia and the positive electrode 1 cm left to the xiphoid. A single injection of carprofen (6 mg/kg s.c.) before starting the surgical procedure and metamizol (300 mg/kg p.o. from 2 days before to 7 days after the surgical procedure) were used for analgesia. Recordings were started after a recovery time of at least two weeks. Each mouse received two intraperitoneal injections: (1) 0.9% NaCl solution and (2) atropine 0.5 mg/kg body mass after heart rate recovery from the first injection. Recording and analysis parameters were set according to the manufacturer’s instructions using Ponemah 5.2 software (DSI). Heart rate is given as the average of 1 min intervals.

### Immunoblot analysis

Transfected PDE4D3 from HEK293 cell lysates was detected using a custom pan-PDE4D antibody raised against the C-terminus of PDE4D (see Ref.^34^). Blots were scanned and analyzed densitometrically by NIH Image J software for uncalibrated optical density.

### Contractility measurements in human atrial trabeculae

Thin human atrial trabeculae (cross section area 0.61±0.08 mm^2^) were dissected from the right atrial appendage of patients (n = 3) with sinus rhythm in accordance with ethical guidelines and approval from the Unversitätsmedizin Göttingen. Isometric force recordings were performed as previously described^38^.

### Statistical analysis

Normal distribution was tested by the Kolmogorov-Smirnov test. Differences between experimental groups were analyzed using Origin software and one-way ANOVA or paired t-test (as appropriate) at the significance level of p < 0.05, followed by Bonferoni’s post-hoc test. Data are presented as means ± s.e. from the indicated numbers of independent experiments (mice or cells) per condition.

### Data availability

All data are included in the manuscript and as supplementary information.

## Electronic supplementary material

Supplementary Information
