# Supplementary material for: Atropine augments cardiac contractility by inhibiting cAMP-specific phosphodiesterase type 4
Source: Sci Rep. 2017 Nov 9;7:15222. doi: 10.1038/s41598-017-15632-x (PMC5680190; doi:10.1038/s41598-017-15632-x)
Supplement: Supplementary file 1 — Supplementary Information [file 41598_2017_15632_MOESM1_ESM.pdf]

ONLINE SUPPLEMENT TO:

Atropine augments cardiac contractility by inhibiting cAMP-specific phosphodiesterase type 4

Ruwan K. Perera,[1,2] Thomas H. Fischer,[2] Michael Wagner,[3] Matthias Dewenter,[3] Christiane Vettel,[4] Nadja I. Bork,[1,5] Lars S. Maier,[6] Marco Conti,[7] Juergen Wess,[8] Ali El-Armouche,[3] Gerd Hasenfuß,[2,5] and Viacheslav O. Nikolaev[1,5]

[1]Institute of Experimental Cardiovascular Research, University Medical Center Hamburg-Eppendorf, Hamburg, Germany

[2]Clinic of Cardiology and Pulmonology, Heart Research Center Göttingen, Georg August University Medical Center, Göttingen, Germany

[3]Institute of Pharmacology and Toxicology, Technical University of Dresden, Dresden, Germany

[4]Institute of Experimental and Clinical Pharmacology and Toxicology, Mannheim Medical Faculty, Heidelberg University, Germany

[5]DZHK, German Center for Cardiovascular Research, partner sites Hamburg and Göttingen

[6]Department of Internal Medicine II, University Hospital Regensburg, Regensburg, Germany

[7]Department of Obstetrics, Gynecology and Reproductive Sciences, University of California, San Francisco, United States

[8]Molecular Signaling Section, Laboratory of Bioorganic Chemistry, National Institute of Diabetes and Digestive and Kidney Diseases (NIDDK), Bethesda, Maryland, USA

Address for Correspondence:

Prof. Dr. Viacheslav Nikolaev
Institute of Experimental Cardiovascular Research
University Medical Center Hamburg-Eppendorf (UKE)
Martinistr. 52
D-20246 Hamburg
Germany
Phone: +49-40-7410-51391; Fax: +49-40-7410-40180
E-mail: v.nikolaev@uke.de

Supplementary Figures

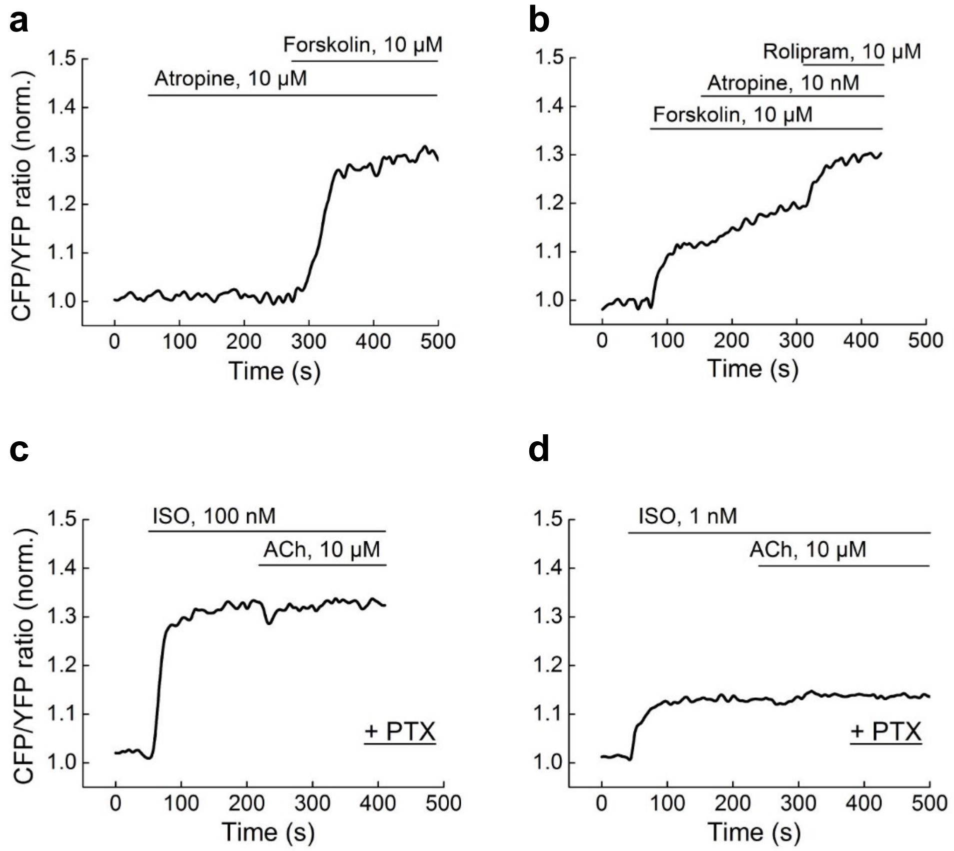

Supplementary Figure 1. Atropine effects on cardiomyocyte cAMP levels. **(a)** Atropine alone, even at a very high concentration (10 μM), does not affect intracellular cAMP levels. Forskolin (10 μM) was used as a positive control. **(b)** Atropine (10 nM) potentiates forskolin-mediated increases in cAMP. The PDE4 inhibitor rolipram (10 μM) was applied to maximally activate the sensor. **(c, d)** Inhibition of G_i-proteins with pertussis-toxin (1.5 μg/ml for 6-8 h) prevents the ACh (10 μM)-mediated cAMP decrease after 100 nM (in *d*) or 1 nM (in *c*) ISO prestimulation. All traces are representative experiments (n=5); pooled data are shown in Figure 1C and 1F.

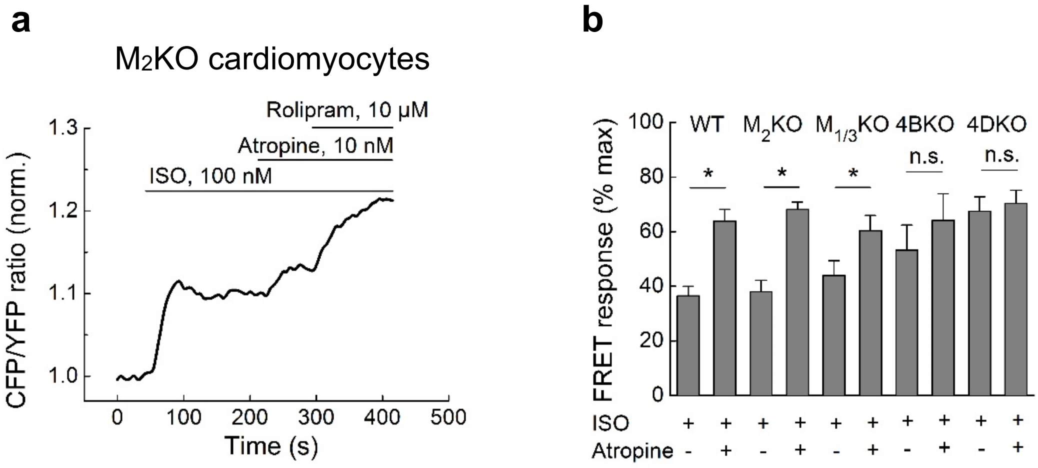

Supplementary Figure 2. Effect of atropine on cAMP levels in cardiomyocytes isolated from M_2- and $M_{1/3}$-receptor knockout mice as well as from PDE4B and PDE4D knockout mice. **(a)** Representative experiment and **(b)** quantification (n=4-11 cells each) are shown. Means ± s.e.m. *differences are statistically significant at $p<0.01$ by paired t-test. n.s – not significant at $p=0.05$. Myocytes were transduced with the cAMP sensor expressing adenovirus and stimulated with 100 nM isoproterenol (ISO) followed by 10 nM atropine and 10 µM rolipram.

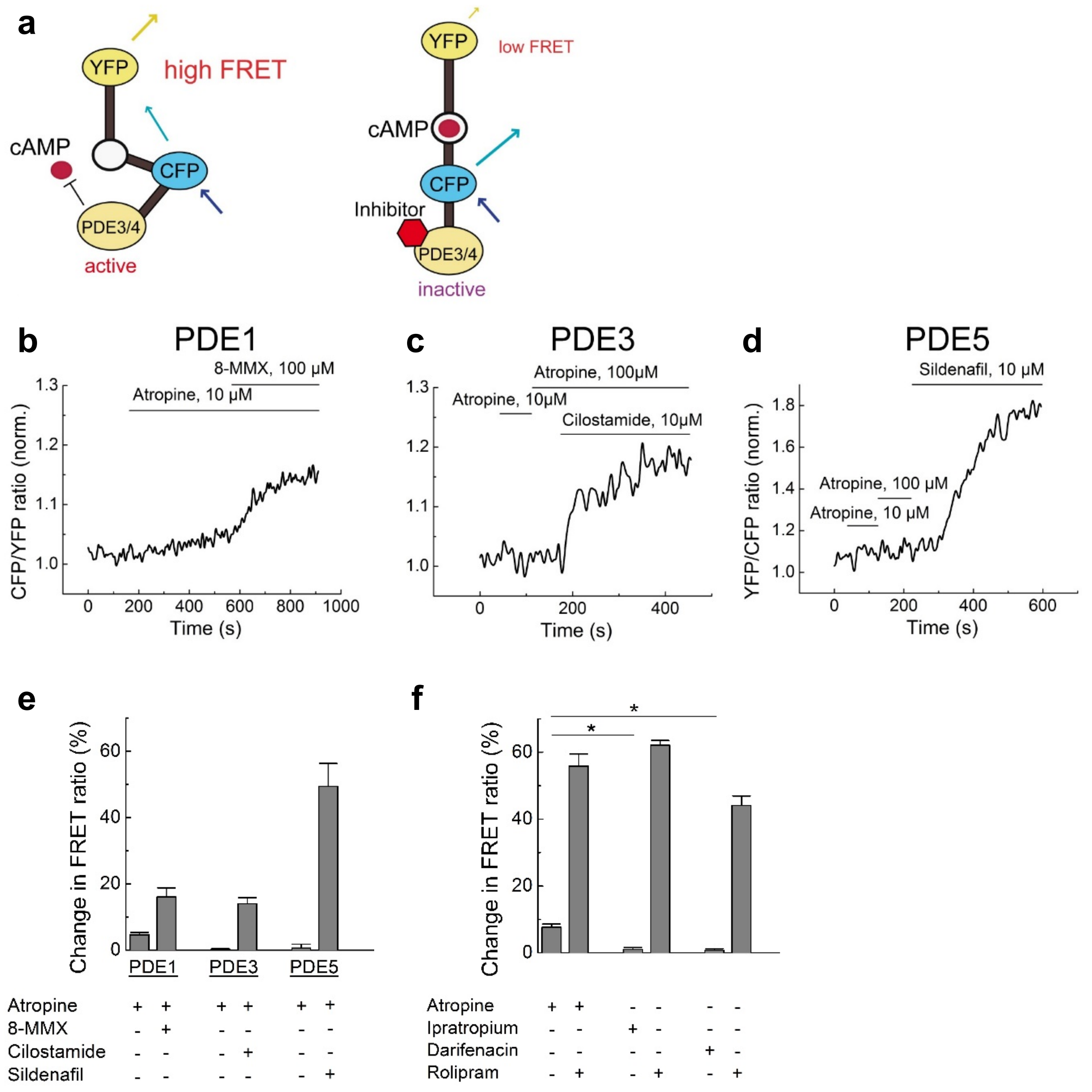

Supplementary Figure 3. Measurements of PDE inhibition in HEK293A cells. **(a)** Schematics of the fusion sensors which monitor cAMP in the vicinity of a PDE isoform of interest and directly report PDE inhibition. Epac1-camps sensor is fused to the N-terminus of a PDE and does not respond to intracellular cAMP due to the constitutive PDE activity. Inhibiting PDE with an inhibitor leads to cAMP binding to the sensor and to an increase in distance between the two fluorophores CFP and YFP. **(b)** Atropine at a very high concentration (10 µM) only slightly inhibits PDE1 activity (8-MMX, 8-methoxymethyl-3-isobutyl-1-methylxanthine, 100 µM) but **(c)** does not affect PDE3. **(d)** Atropine does not inhibit PDE5 activity in cells expressing the cGES-DE2-PDE5A sensor. Cells were prestimulated with 1 µM ISO (in *B-C*) or with 50 µM sodium nitroprusside (in *d*) for 3 min before adding atropine to pre-elevate intracellular cAMP and cGMP levels, respectively. **(e)** Quantification of the data shown in *b-d* as a % change of the FRET ratio in response to atropine for each individual PDE-sensor along with maximal effects measured by these sensors with respective inhibitors, means ± s.e.m. (n=6). **(f)** Inhibition of PDE4 by various anticholinergic drugs monitored in HEK293 cells stably expressing the Epac1-camps-PDE4 sensor. Measurements were performed as described above with 10 nM atropine and 1 µM ipratropium or 1 µM darifenacin. Means ± s.e.m. (n=8 each), * $p<0.05$ by one-way ANOVA.

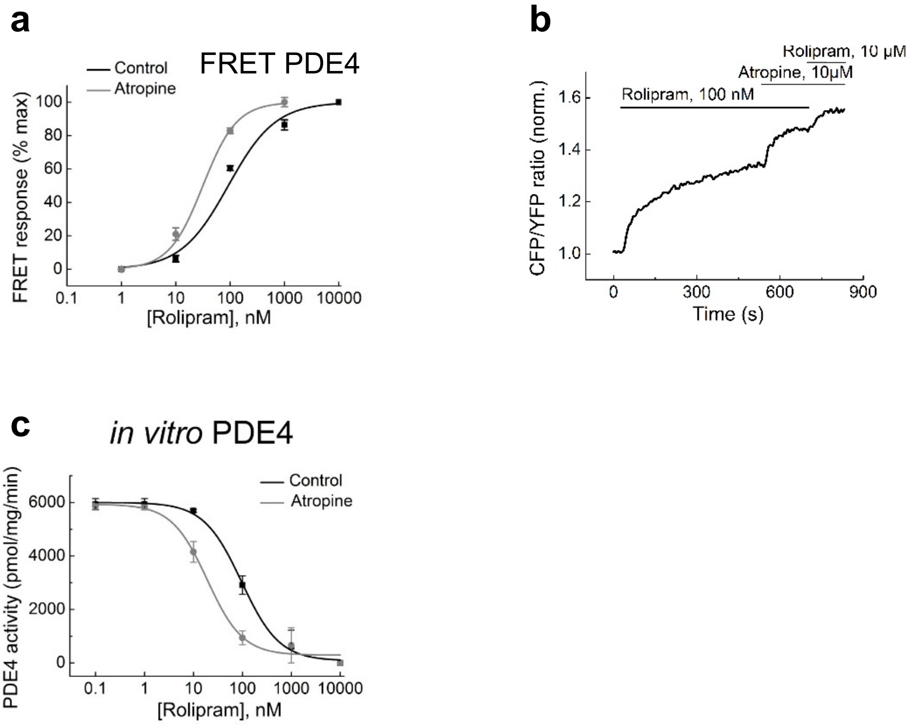

Supplementary Figure 4. Atropine inhibits PDE4 in an allosteric manner. **(a)** Concentration-response-dependencies for rolipram measured using the Epac1-camps-PDE4A sensor in the presence or absence of 10 µM atropine (10 min pre-incubation without ISO), means ± SE (n=6-8). The IC_{50}-values were 29.9 ± 1.4 nM and 72.6 ± 5.7 nM with and without atropine, respectively (P<0.01 by one-way ANOVA). **(b)** Representative FRET trace (n=6) showing a “shift” of rolipram efficacy at the sensor induced by atropine. **(c)** Measurements of PDE4A activity (recombinant protein) and its inhibition by rolipram *in vitro* in absence and presence of 10 µM atropine. The IC_{50}-values were 19.1 ± 4.8 and 96.1 ± 11.2 nM with and without atropine, respectively. Data are means ± s.e.m. (n=3). P<0.01 by one-way ANOVA. This behavior is in line with the effects of allosteric modulators of PDE4 activity[1,2].

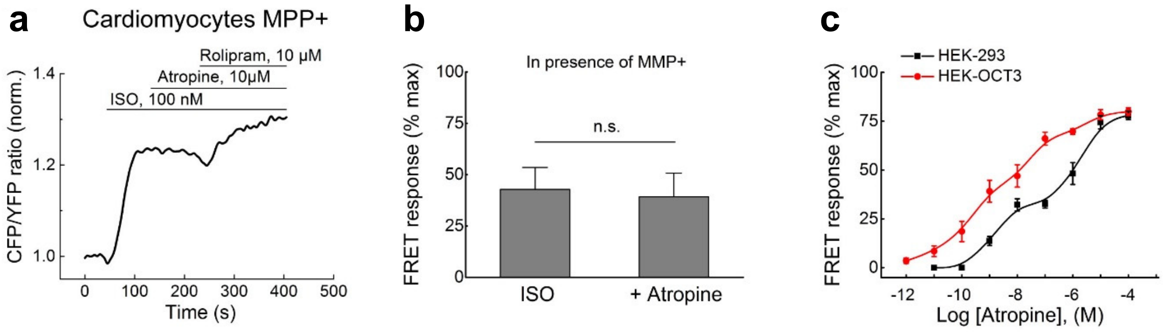

Supplementary Figure 5. Organic cation transporters (OCTs) might be involved in the intracellular effects of atropine. **(a)** Preincubation of cardiomyocytes with the OCT inhibitor MPP+ (100 µM) prevents the stimulatory effect of atropine measured as in Figure 1b. Representative trace, n=5. Quantification is shown in **b**. n.s. – not significant. **(c)** Stable overexpression of OCT3 in HEK293 cells potentiates the stimulatory effect of atropine measured as in Figure 2A, means ± s.e.m. (n=5).

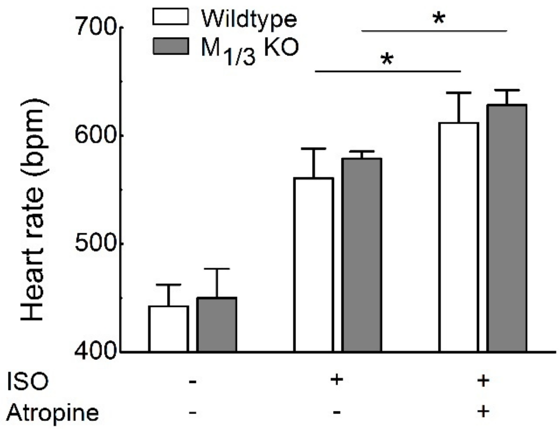

Supplementary Figure 6. Heart rate measurements in wildtype and $M_{1/3}$-receptor knockout Langendorff-perfused hearts stimulated with ISO alone (10 nM) or with ISO plus atropine (10 nM). Atropine applied after ISO significantly increases the beating frequency in both genotypes. Shown are means ± s.e.m. (n=4-6). * $p<0.05$, by paired t-test.

Supplementary References

1 Bolger, G. *et al.* Attenuation of the activity of the cAMP-specific phosphodiesterase PDE4A5 by interaction with the immunophilin XAP2. *J. Biol. Chem.* **278**, 33351–33363 (2003).

2 Burgin, A. B. *et al.* Design of phosphodiesterase 4D (PDE4D) allosteric modulators for enhancing cognition with improved safety. *Nat. Biotechnol.* **28**, 63-70 (2010).
